# Comparative study of clinical and MRI features of TMD patients with or without joint effusion: a retrospective study

**DOI:** 10.1186/s12903-024-04065-4

**Published:** 2024-03-09

**Authors:** Chuanjie Li, Benyi Chen, Rong Zhang, Qingbin Zhang

**Affiliations:** 1 Department of Temporomandibular Joint Surgery, Guangdong Engineering Research Center of Oral Restoration and Reconstruction, Guangzhou Key Laboratory of Basic and Applied Research of Oral Regenerative Medicine, Affiliated Stomatology Hospital of Guangzhou Medical University, Guangzhou, 510000 China; 2 School of Stomatology, Guangzhou Medical University, Guangzhou, China

**Keywords:** Disc morphology, Disc position, Condylar bone, Bone marrow oedema, Joint effusion

## Abstract

**Background:**

The relationship between joint effusion and temporomandibular disorders (TMD) remains unclear. The purpose of this study was to investigate the correlation among joint effusion, clinical features and MRI imaging features of TMD.

**Methods:**

A total of 1532 temporomandibular joints (TMJs) from 766 patients (605 females and 161 males) with the mean age of 31.68 ± 13.71 years from January 2022 to June 2023 were included in the study. Clinical and MRI features were collected and analyzed. Chi-Square test, Spearman correlation coefficient and binary logistic regression analysis were performed.

**Results:**

Patients with joint effusion were significantly older and had smaller value of MIO (*p* < 0.001). There were significant differences in the distribution of joint sounds (with or without), joint pain (with or without), disc morphology (biconcave, contracture, irregular and lengthened) and disc position between joint effusion group (JE) and non-joint effusion group (NA) (*P* < 0.05).The odds of having joint effusion were 1.726 higher in patients with joint sounds when compared to those without joint sounds. The odds of having joint effusion were 8.463 higher in patients with joint pain when compared to those without joint pain. The odds of having joint effusion were 2.277 higher in patients with contracture when compared to those with biconcave. The odds of having joint effusion were 1.740 higher in patients with anterior disc displacement with reduction (ADDWR) when compared to those with normal disc position. The prediction accuracy of this model is 74.9%, and the area under curve (AUC) is 79.5%, indicating that it can be used for the prediction and the judgment effect is average.

**Conclusions:**

The results demonstrated that joint sounds, joint pain, contracture, and ADDWR are high risk factors for joint effusion, especially joint pain.

**Trial registration:**

This study was retrospectively registered on 28/03/2022 and endorsed by the Ethics Committee of Affiliated Stomatology Hospital of Guangzhou Medical University (LCYJ2022014).

## Background

Temporomandibular disorders (TMD) are a group of disorders involving the temporomandibular joint (TMJ), masticatory muscles, and related bone and soft tissues, usually characterized by clinical symptoms such as joint sounds, pain, and limited mouth opening. Standard clinical examination and necessary imaging examination are the key to the diagnosis of TMD.

Magnetic resonance imaging (MRI) of the temporomandibular joint (TMJ) is a well-established diagnostic imaging technique that can provide detailed anatomical information, such as disc morphology, disc position, condylar morphology and joint effusion, and is currently considered as a important complementary exam for the diagnosis of TMD [1].

In consideration that some patients with abnormal MRI images have no obvious clinical symptoms, or some patients with obvious TMD symptoms have no obvious imaging changes, scholars have carried out several studies on the correlation between clinical manifestations and imaging features. Among them, many studies focused on the correlation between disc morphology, disc position or condylar morphology and clinical symptoms, but few studies on joint effusion [2–5]. And our previous research suggested that the imaging findings of TMJs were significantly different between symptomatic and asymptomatic TMJs [6].

Synovial fluid of TMJ is the dialysate of plasma, which can provide nutrients such as lipids, cholesterol, phospholipids and hyaluronic acid for TMJs, help remove the waste products [7], lubricate the TMJs and also affect the pressure distribution. Many studies at the cellular and molecular level have demonstrated that synovial fluid plays an important role in the pathogenesis of TMD [8]. If a high signal intensity in the TMJ is seen in T2-weighted sequences of MRI, it is defined as a joint effusion [9]. The quantification of joint effusion in MRI is difficult, so the reliability and validity of the measurement is questionable. The definition of joint effusion varies from study to study. Some are based on the number of high signal continuous layers involved, and some are based on the width of the fluid accumulation [10, 11]. Vogl et al. [3] divided the possible signals of fluid into no signals, signals of fluid and joint effusion.

The association among joint effusion, clinical symptoms of TMD and other MRI images of TMJ is controversial. Although the classification of joint effusion varies, these studies all suggest that effusion was obviously related to ADDwoR [1, 12, 13]. In addition, the incidence of joint effusion is significantly higher in patients suffering from the joint pain and TMJ arthritis than in healthy volunteers [14, 15]. However, Manfredini et al. [16] concluded that the relationship between the cause of disc displacement and joint effusion is not clear. Studies revealing the association among joint effusion, other clinical manifestations and disc morphology, etc., are seldom and lack large sample sizes.

Since the relationship between joint effusion and TMD remains unclear, the main objective of this retrospective study was to investigate the correlation between joint effusion and clinical features and MRI imaging features of TMD patients, aiming to providing a reference for clinical treatment of TMJ effusion.

## Methods

### Study design

This study was endorsed by the Ethics Committee of the Affiliated Stomatology Hospital of Guangzhou Medical University (LCYJ2022014). The need for written informed consent was waived because of the retrospective nature of this study. Eligible patients admitted to the TMJ Diagnosis and Treatment Center from January 2022 to June 2023 were recruited in the study. MRI images of the TMJ region were accrued from consecutive new patients in the imaging center. Clinical data from the electronic health records of corresponding patients were retrieved to evaluate the relationship among joint effusion, clinical symptoms and other MRI characteristics. All participants received clinical examination according to Diagnostic Criteria for TMDs (DC/TMD) methodology. Cases with high-quality of MRI images and sufficient valid clinical data were included, and those with maxillofacial trauma and tumor, prior TMJ surgery, orthodontics treatment, ankylosis, severe morphological abnormalities of TMJ and poor quality scanning images were excluded.

### Simple random sampling

Simple random sampling in IBM SPSS Statistics 23 software was used to select participants for this study. The specific process is as follows: (1) Set random seeds: open the Random Number Generator and set the seed to 20,230,914. (2) Set numeric expression: open the Compute and set the Numeric Expression to RV.UNIFORM (0, 100). (3) All data generates a corresponding random number. (4) Random numbers are sorted from small to large. (5) According to the required sample size, select the corresponding research objects in front.

**Fig. 1:**
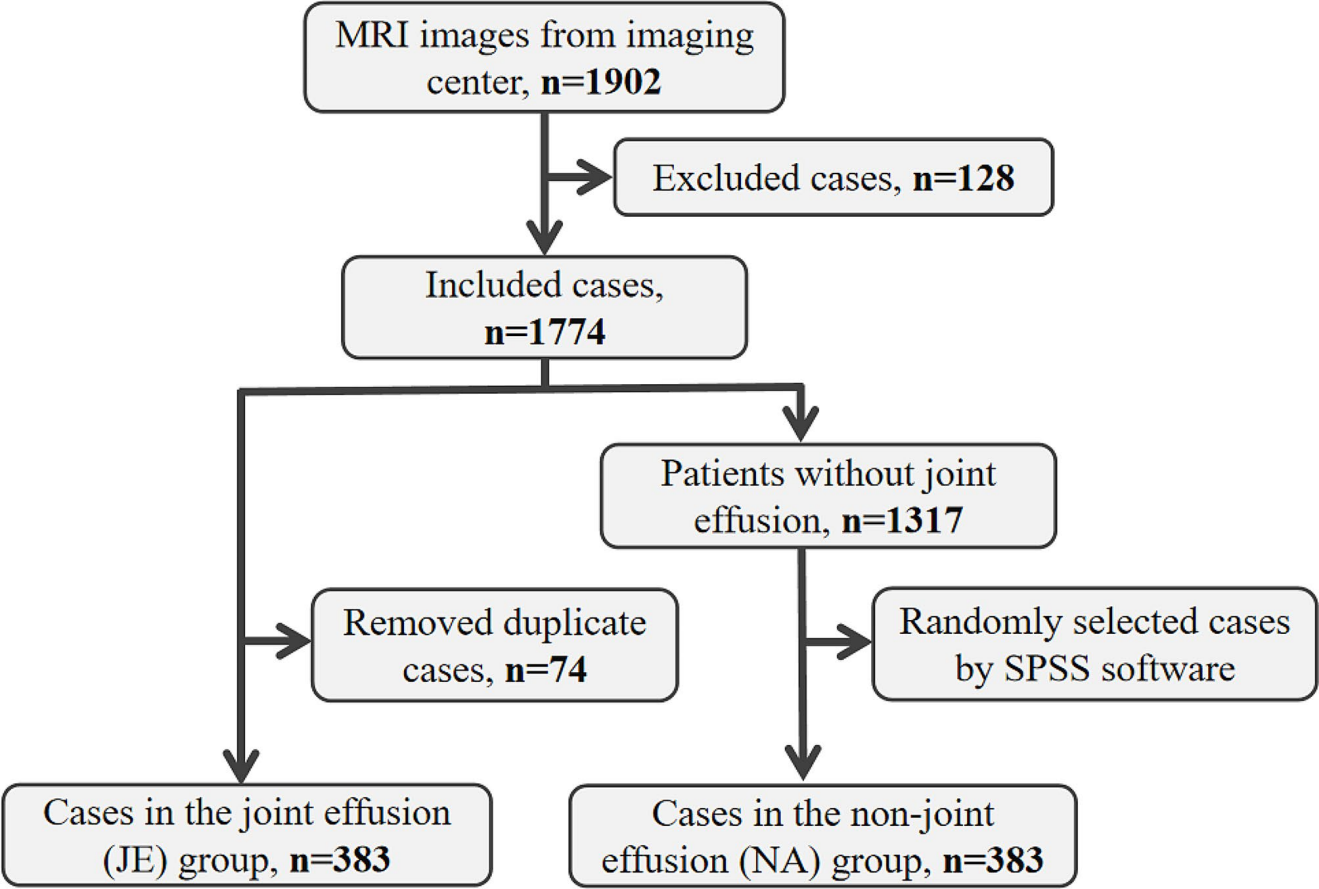
Flow diagram of the case selection process

A total of 1532 TMJs from 766 participants (605 females and 161 males) with the mean age of 31.68 ± 13.71 years were brought into the study. The flow diagram of the case selection process was shown in Fig. 1. All the cases were divided into 2 groups: the joint effusion (JE) group and the non-joint effusion (NA) group.

### Clinical examination

All patients underwent professional clinical examination according to DC/TMD Axis I. Clinical examination results like joint sounds, joint pain, and maximal interincisal opening (MIO) are recorded in the electronic medical records. The clinical examination data obtained in this study are as the follows. Joint sounds and joint pain were recorded by palpation around the TMJ region. Patients were guided to perform jaw movement, including opening and closing movement, forward and backward movement and lateral movement 3 times respectively. In terms of joint sounds, clicking, popping, and crepitation were recorded as abnormal. Cases with joint pain were recorded as abnormal. The distance between the upper and lower incisors when opening the mouth as wide as possible was recorded as MIO.

### Magnetic resonance imaging

MRI images of bilateral TMJs were obtained at closed and wide open mouth positions by a 1.5T MR Imaging scanner (UNITED IMAGING uMR, China). The patients were supine and wore ear plugs to protect their ears. In the closed-mouth position, the TMJ oblique sagittal plane and the coronal plane were captured when patients were in the intercuspal position. In the open-mouth position, the TMJ oblique sagittal plane were captured when patients were asked to open the mouth as wide as possible and bite a mouth opener. T1 weighted images (T1WI), T2 weighted images (T2WI) and proton-density weighted images (PDWI) were scaned. The field-of-view (FOV) was set to 140 × 140 mm, with the section of thickness (Thk) set to 2.5 mm. Averagely, 9 images each were taken for each TMJ, 30 min per patient.

### MRI diagnosis

#### Disc morphology

Based on oblique sagittal PDWI at closed-mouth position, the disc morphology was divided into four types according to the degree of folding [6]. (1) Biconcave: the normal articular disc with narrowed intermediate zone and fully visible posterior and anterior bands. (2) Lengthened: the articular disc with equal thickness of the anterior, intermediate, and posterior band. (3) Contracture: the articular disc with contracture shape. (4) Irregular: the articular disc with irregular strip or nodules disc shape, or the disc is missing in shape and the signal is discontinuous.

#### Disc position

Based on oblique sagittal adiposity-suppressed T2WI both at closed- and open-mouth position, the disc displacement is divided into 5 types [6]. (1) The posterior band of the disc located directly superior to the condylar head in the closed position (between 11 and 12 o’clock) is defined as the normal disc position (NA). (2) The posterior band of the disc located anterior to the condylar head in the closed position (< 11 o’clock), and turned normal in the open position is defined as anterior disc displacement with reduction (ADDWR). (3) The posterior band of the disc located anterior to the condylar head in the closed position (< 11 o’clock), and did not turn normal in the open position is defined as anterior disc displacement without reduction (ADDWoR). (4) The posterior band of the disc located posterior to the condylar head in the closed position (> 1 o’clock), and turned normal in the open position is defined as posterior disc displacement with reduction (PDDWR). (5) The posterior band of the disc located posterior to the condylar head in the closed position (> 1 o’clock), and did not turn normal in the open position is defined as posterior disc displacement without reduction (PDDWoR).

#### Joint effusion

Based on oblique sagittal adiposity-suppressed T2WI at closed-mouth position, the joint effusion was divided into two types according to the liquid signal. (1) No joint effusion (NA). (2) The liquid signal involving more than 4 consecutive layers of images is defined as joint effusion.

#### Condylar bone morphology

Based on oblique sagittal adiposity-suppressed T2WI at closed-mouth position, the condylar bone morphology is divided into normal and abnormal. The presence of erosion, destruction, osteophyte, sclerosis or cystic changes on the articular surface was defined as the abnormal.

#### Bone marrow oedema

Bone marrow edema is diagnosed when condylar bone marrow exhibited hyperintense changes on oblique sagittal PDWI and hypointense changes on T1WI at closed-mouth position. The presence/absence of bone marrow oedema was recorded.

### Establishment of binary logistic regression model

Since the Chi-square test of joint sounds, joint pain, disc morphology and disc position had statistical significance, a binary Logistic regression model was established for these four categories. The presence and absence of joint effusion were taken as the dependent variables, and the Logistic regression values were defined as no joint effusion = 0 and joint effusion = 1, respectively. The following features were used as independent variables to establish a Logistic model (including criteria *P* < 0.05 and excluding criteria *P* > 0.1). The forward method was used for stepwise regression. Criteria for assigning values: joint sounds (normal = 0 and abnormal = 1); joint pain (normal = 0 and abnormal = 1); disc morphology (biconcave = 0, lengthened = 1, contracture = 2 and irregular = 3); disc position (normal = 0, ADDWR = 1, ADDWoR = 2, PDDWR = 3 and PDDWoR = 4).

### Data analysis

SPSS Statistics 23.0 software was used for statistical analysis of the eligible data. The mean age and MIO were described by mean ± standard deviation. Chi-Square test was performed for the distribution of sex, joint sounds, joint pain, disc morphology, disc position, joint effusion, condylar bone morphology, and bone marrow oedema. Pearson Chi-Square test was performed for the expected frequency of less than 5. Spearman correlation coefficient was performed to assess the correlations between the study variables. Single-factor binary logistic regression analysis was used to initially screen out the variables that might be meaningful. The preliminary screening boundary value was *P* < 0.1. If the difference of the factor was statistically significant (*P* < 0.1), such indicator was included in multi-factor binary logistic regression analysis to further verify the effect and estimate the effect size. The receiver operating curve (ROC) was used to assess the predicting accuracy of this model. *P* < 0.05 was considered statistically significant.

## Results

### Basic characteristics

In this study, 383 patients with joint effusion were included in joint effusion group (JE), and another 383 eligible patients which were randomly selected by SPSS Statistics 23.0 software were included in non-joint effusion group (NA). Secondly, as shown in Table 1, the 383 patients with joint effusion were divided into two groups according to the joint effusion side: joint effusion on one side (1JE) group (*n* = 308) and joint effusion on two-side (2JE) group (*n* = 75). Female ratio, the mean age and MIO were studied among 1JE (*n* = 308), 2JE (*n* = 75), and NA (*n* = 383) (Table 1). There was no significant difference in gender distribution (χ2 = 4.388, *P* = 0.111). There were significant differences in age and MIO among the three groups (F = 116.651, *P* = 0.000 and F = 897.720, *P* = 0.000, respectively). Patients with joint effusion were older and had less mouth opening, especially in 2JE group.

**Table 1:** Basic characteristics of the included patients

	1JE (*n* = 308)	2JE (*n* = 75)	NA (*n* = 383)	*P* value
Female n (%)	251 (81.5)	63 (84.0)	291 (76.0)	χ2 = 4.388, *P* = 0.111
Age (mean ± SD)	32.98 ± 13.83	33.46 ± 16.78	30.18 ± 12.71	F = 116.651, *P* = 0.000
MIO (mean ± SD)	37.39 ± 9.10	36.98 ± 8.91	42.25 ± 7.55	F = 897.720, *P* = 0.000

Female ratio was obtained from Pearson Chi-Square test analysis and the mean age and MIO were obtained from one-way ANOVA. Where: MIO = maximal interincisal opening. Significant difference was set at *P* < 0.05.

### Comparison between the JE side and NA side of TMJs

Firstly, we divided 1532 TMJs into 2 groups according to whether or not there was a joint effusion: JE side (*n* = 458) and NA side (*n* = 1074). The distribution and the indirect relation were performed by Pearson Chi-Square test and Spearman correlation coefficient respectively, as shown in Table 2.

There were significant differences in the distribution of joint sounds (χ2 = 22.351, *P* = 0.000), joint pain (χ2 = 345.874, *P* = 0.000), disc morphology (χ2 = 77.872, *P* = 0.000), disc position (χ2 = 75.319, *P* = 0.000) and condylar bone morphology (χ2 = 10.063, *P* = 0.002) in the JE side group and NA side group (*P* < 0.05), and showed positively correlated with joint effusion (*P* < 0.05). There was no significant difference in the distribution of condylar bone marrow edema in the JE side group and NA side group (χ2 = 0.227, *P* = 0.634).

The proportion of joint sounds in the NA side group was higher than that in the JE side group. Patients without joint pain ranked first in the NA side group, while patients with joint pain ranked first in the JE side group. Both in the NA side and JE side group, contracture ranked first followed by biconcave, irregular, lengthened; and ADDWoR ranked first followed by ADDWR, NA, PDDWR, PDDWoR. Patients with normal condylar bone morphology ranked first in the NA side group, while patients with abnormal condylar bone morphology ranked first in the JE side group.

**Table 2:** Comparison between all the JE and NA TMJs

		NA side (*n* = 1074)	JE side (*n* = 458)	*P* value	r, P
Joint sounds	normal (%)	770 (71.7)	272 (59.4)	χ2 = 22.351, *P* = 0.000	*r* = 0.121, *P* = 0.000
	abnormal (%)	304 (28.3)	186 (40.6)		
Joint pain	normal (%)	889 (82.8)	158 (34.5)	χ2 = 345.874, *P* = 0.000	*r* = 0.475, *P* = 0.000
	abnormal (%)	185 (17.2)	300 (65.5)		
Disc morphology	biconcave (%)	389 (36.2)	84 (18.3)	χ2 = 77.872, *P* = 0.000	*r* = 0.154, *P* = 0.000
	lengthened (%)	118 (11.0)	33 (7.2)		
	contracture (%)	415 (38.6)	285 (62.2)		
	irregular (%)	152 (14.2)	56 (12.2)		
Disc position	NA (%)	282 (26.3)	46 (10.0)	χ2 = 75.319, *P* = 0.000	*r* = 0.216, *P* = 0.000
	ADDWR (%)	323 (30.1)	111 (24.2)		
	ADDWoR (%)	452 (42.1)	291 (63.5)		
	PDDWR (%)	13 (1.2)	6 (1.3)		
	PDDWoR (%)	4 (0.4)	4 (0.9)		
Condylar bone	normal (%)	606 (56.4)	218 (47.6)	χ2 = 10.063, *P* = 0.002	*r* = 0.081, *P* = 0.001
	abnormal (%)	468 (43.6)	240 (52.4)		
Bone marrow oedema	absence (%)	1058 (98.5)	448 (97.8)	χ2 = 0.926, *P* = 0.336	-
	presence (%)	16 (1.5)	10 (2.2)		

Where: NA = normal; ADDWR = anterior disc displacement with reduction; ADDWoR = anterior disc displacement without reduction; PDDWR = posterior disc displacement with reduction; PDDWoR = posterior disc displacement without reduction. Significant difference was set at *P* < 0.05.

In order to prevent errors caused by uneven sample sizes, another 458 TMJs were randomly selected from 766 TMJs without joint effusion from NA side group by SPSS software as the control group, as shown in Table 3. The distribution and the indirect relation were performed by Pearson Chi-Square test and Spearman correlation coefficient respectively. There were significant differences in the distribution of joint sounds (χ2 = 4.200, *P* = 0.040), joint pain (χ2 = 190.960, *P* = 0.000), disc morphology (χ2 = 70.635, *P* = 0.000) and disc position (χ2 = 53.983, *P* = 0.000) in the JE side group and NA side group (*P* < 0.05), and showed positively correlated with joint effusion (*P* < 0.05). There were no significant differences in the distribution of condylar bone morphology and condylar bone marrow edema in the JE side group and NA side group (χ2 = 1.262, *P* = 0.261 and χ2 = 0.227, *P* = 0.634).

Differences from the above statistical results include the followings: (1) In the NA side group, biconcave ranked first followed by contracture, irregular and lengthened. (2) In the JE side group, contracture ranked first followed by biconcave, irregular and lengthened. (3) In the NA side group, ADDWoR ranked first followed by NA, ADDWR, PDDWR and PDDWoR. (4) In the JE side group, ADDWoR ranked first followed by ADDWR, NA, PDDWR and PDDWoR.

In order to avoid the interference of non-joint effusion TMJs from patients with unilateral joint effusion, we finally randomly selected 458 TMJs from 766 TMJs in patients without joint effusion as the control group for the follow-up study.

**Table 3:** Comparison between 458 JE and 458 NA TMJs

		NA side (*n* = 458)	JE side (*n* = 458)	*P* value	r, P
Joint sounds	normal (%)	302 (65.9)	272 (59.4)	χ2 = 4.200, *P* = 0.040	*r* = 0.068, *P* = 0.040
	abnormal (%)	156 (34.1)	186 (40.6)		
Joint pain	normal (%)	365 (79.7)	158 (34.5)	χ2 = 190.960, *P* = 0.000	*r* = 0.457, *P* = 0.000
	abnormal (%)	93 (20.3)	300 (65.5)		
Disc morphology	biconcave (%)	164 (35.8)	84 (18.3)	χ2 = 70.635, *P* = 0.000	*r* = 0.157, *P* = 0.000
	lengthened (%)	57 (12.4)	33 (7.2)		
	contracture (%)	160 (34.9)	285 (62.2)		
	irregular (%)	77 (16.8)	56 (12.2)		
Disc position	NA (%)	126 (27.5)	46 (10.0)	χ2 = 53.983, *P* = 0.000	*r* = 0.225, *P* = 0.000
	ADDWR (%)	121 (26.4)	111 (24.2)		
	ADDWoR (%)	203 (44.3)	291 (63.5)		
	PDDWR (%)	6 (1.3)	6 (1.3)		
	PDDWoR (%)	2 (0.4)	4 (0.9)		
Condylar bone	normal (%)	235 (51.3)	218 (47.6)	χ2 = 1.262, *P* = 0.261	-
	abnormal (%)	223 (48.7)	240 (52.4)		
Bone marrow oedema	absence (%)	450 (98.3)	448 (97.8)	χ2 = 0.227, *P* = 0.634	-
	presence (%)	8 (1.7)	10 (2.2)		

Where: NA = normal; ADDWR = anterior disc displacement with reduction; ADDWoR = anterior disc displacement without reduction; PDDWR = posterior disc displacement with reduction; PDDWoR = posterior disc displacement without reduction. Significant difference was set at *P* < 0.05.

### Risk factors for joint effusion of TMJs

Firstly, the single-factor binary logistic regression analysis was conducted between the NA side and JE side group. The preliminary screening boundary value was *P* < 0.1. Statistical analysis of PDDWR and PDDWoR was not performed because of the small number of cases with PDD according to clinical knowledge. The overall differences of joint sounds, joint pain, disc morphology and disc position were statistically significant (*P* < 0.1) (Table 4), such indicators were then included in multi-factor binary logistic regression analysis to further verify the effect and estimate the effect size (Table 5). Hosmer-Lemeshow (HL) test was performed for the model goodness of fit. The result was *P* = 0.231, which indicated that the data information was fully extracted and the model showed a high goodness of fit. The prediction accuracy of this model was 74.9%.

**Table 4:** Single-factor binary logistic regression analysis

		OR	CI (95%, Lower-Upper)	*P* value
	Joint sounds	1.324	1.012–21.732	0.041
	Joint pain	7.452	5.530-10.042	0.000
Disc morphology	biconcave			0.000
	lengthened	1.130	0.684–1.869	0.633
	contracture	3.478	2.509–4.821	0.000
	irregular	1.420	0.921–2.190	0.113
Disc position	NA			0.000
	ADDWR	2.513	1.643–3.842	0.000
	ADDWoR	3.927	2.679–5.755	0.000

Where: NA = normal; ADDWR = anterior disc displacement with reduction; ADDWoR = anterior disc displacement without reduction; PDDWR = posterior disc displacement with reduction; PDDWoR = posterior disc displacement without reduction. OR: odds ratio, CI: confidence interval.

**Table 5:** Multi-factor binary logistic regression analysis

		OR	CI (95%, Lower-Upper)	*P* value
	Joint sounds	1.726	1.254–2.378	0.001
	Joint pain	8.463	6.026–11.886	0.000
Disc morphology	biconcave			0.000
	lengthened	0.734	0.402–1.340	0.314
	contracture	2.277	1.389–3.732	0.001
	irregular	0.806	0.431–1.507	0.499
Disc position	NA			0.016
	ADDWR	1.740	1.044–2.901	0.034
	ADDWoR	1.188	0.650–2.169	0.575

Where: NA = normal; ADDWR = anterior disc displacement with reduction; ADDWoR = anterior disc displacement without reduction; PDDWR = posterior disc displacement with reduction; PDDWoR = posterior disc displacement without reduction. OR: odds ratio, CI: confidence interval.

In Multi-factor binary logistic regressions, the four factors were found to be statistically significant: joint sounds [*P* = 0.001, OR = 1.726, CI(95%)=(1.254–2.378)], joint pain [*P* = 0.000, OR = 8.463, CI(95%)=(6.026–11.886)], contracture [*P* = 0.001, OR = 2.277, CI(95%)=(1.389–3.732)], and ADDWR [*P* = 0.001, OR = 1.740, CI(95%)=(1.044–2.901)].

The odds of having joint effusion were 1.726 higher in persons with joint sounds when compared to those without joint sounds. The odds of having joint effusion were 8.463 higher in persons with joint pain when compared to those without joint pain. The odds of having joint effusion were 2.277 higher in persons with contracture disc morphology when compared to those with biconcave. The odds of having joint effusion were 1.740 higher in persons with ADDWR when compared to those with normal disc position. There was no statistically significant difference in the risk of joint effusion in patients with lengthened and irregular disc morphology compared with patients with biconcave (*P* = 0.314 and *P* = 0.499 respectively). There was no statistically significant difference in the risk of joint effusion in patients with ADDWoR disc position compared with patients with normal disc position (*P* = 0.575).

### ROC curve analysis of the model

**Fig. 2:**
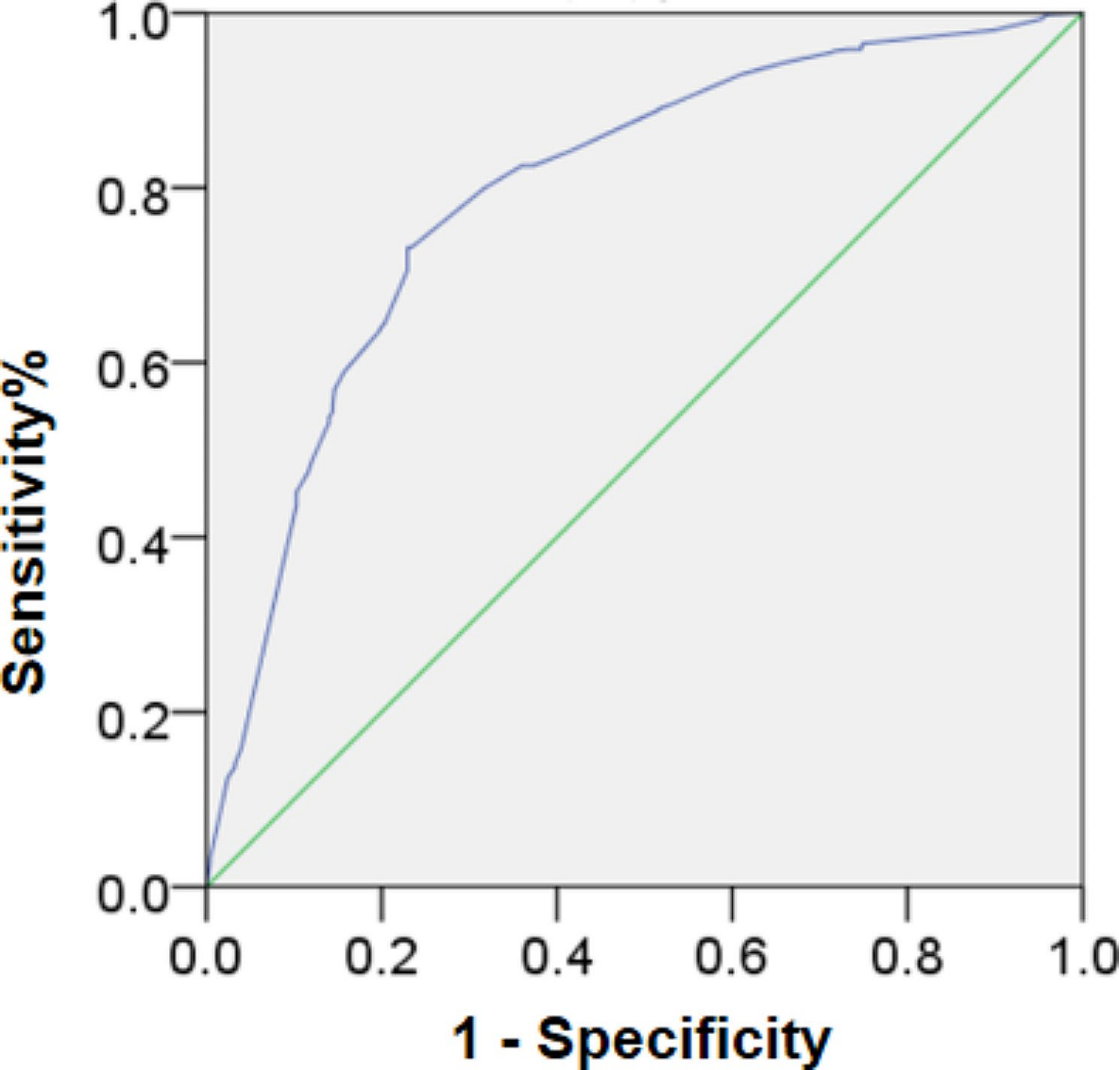
ROC curve analysis of the model

According to the results of Multi-factor binary logistic regression analysis, the ROC curve was further constructed to evaluate the diagnostic value of the above indexes in joint effusion. The area under the curve (AUC) (defined as the area under the ROC curve surrounding the axis) was 79.5%, indicating that the judgment effect of this model was average and could be used to predict the occurrence of TMJ joint effusion (Fig. 2).

## Discussion

In this study, we retrospectively selected 383 patients with joint effusion from 1902 patients who had MRI images, with the incidence of 20.1%. The statistical results showed that joint sounds, joint pain, contracture and ADDWR were high risk factors for joint effusion, and the OR value of joint pain was as high as 8.463.

It is believed that there are two possible mechanisms for joint effusion [13, 17]: Firstly, disc displacement may lead to abnormal mechanical stress, inducing the production of inflammatory mediators and resulting in joint effusion. Secondly, the abnormal disc morphology may interfere with the physiological circulation of synovial fluid, the synovial fluid may accumulate in the joint space.

Joint sound is one of the most common symptoms of TMD, including clicking, popping, crepitation, frictional sound, crushing sound, or murmurs of joints, which are mainly caused by disc displacement and also by the abnormal joint fluid, joint space and condyle. To our knowledge, previous studies [18, 19] determined the possible relationship between disc displacement and degenerative joint disease, but there has been a lack of studies concerning joint sound and joint effusion. In the present study, there was significant difference in the distribution of joint sounds in patients with and without joint effusion, and the odds of having joint effusion were 1.726 higher in patients with joint sounds when compared to those without joint sounds. Abnormal mechanical stress during the joint sounds may aggravate the excessive production of synovial fluid.

TMDs are divided into two categories according to DC/TMD,i.e., painful diseases and TMJ diseases [20, 21]. Joint pain can be affected by jaw movement, function, parafunction, or provocation testing of the TMJ. The relationship between joint pain and intra-articular disorders such as disc displacement and osteoarthritis has been proven [22–24], and many studies have also proved the correlation between joint effusion and joint pain in patients with TMD [10, 25]. In the present study, there was obvious difference in the distribution of joint pain in patients with and without joint effusion. Joint pain was the highest OR among the risk factors included in this study.

The disc morphology has been classified as diverse by different scholars [26, 27]. In our study, the disc morphology was categorised as biconcave, lengthened, contracture, and irregular [28]. We found that the distribution of joint effusion in symptomatic and asymptomatic TMJs was distinct and showed positively correlated with symptoms [6]. The difference in classification makes it difficult to compare different studies results. In the present study, there was significant difference in the distribution of disc morphology in patients with and without joint effusion, and the odds of having joint effusion were 2.277 higher in patients with contracture when compared to those with biconcave, while lengthened and irregular showed no significantly different from biconcave. We speculate that it may be related to the incidence of contracture. When we selected 1074 TMJs without joint effusion as the control group, contracture accounted for about 38.6%, and when we selected 458 TMJs without joint effusion as the control group, contracture accounted for about 34.9%. It should be noted that the contracture of TMJs with joint effusion accounted for 62.2%.

Disc displacement is the most widely studied issue in TMJ internal derangement and is considered to be the main cause of TMD. Consistent with Afroz’s findings [29], the prevalence rate of PDD in TMD patients is as low as about 1.8% (27/1532) in this study. In the present study, there was significant difference in the distribution of disc position in patients with and without joint effusion, and the odds of having joint effusion were 1.740 higher in patients with ADDWR when compared to those with normal disc position. Intriguingly, in our study no obvious difference was found in the risk of joint effusion in patients with ADDWoR comparing with those with normal disc position, which is inconsistent with other studies [1, 13]. This may be related to the different sample size included. In the study of Roh et al. [13], ADDWR and ADDWoR accounted for 30.1% and 23.8% in the non joint effusion group, but 34.7% and 47.3% in the joint effusion group. In the resent study, ADDWR and ADDWoR accounted for 26.4% and 44.3% in the non joint effusion group, while 24.2% and 63.5% in the joint effusion group.

In this study, condylar bone abnormalities were not considered as a risk factor for joint effusion. The possible reason was that the proportion of condylar bone abnormalities was not significantly different between the patients with and without joint effusion, especially when the sample size of TMJ without joint effusion was 458. Meanwhile, bone marrow edema has been shown to be associated with TMJ pain, but there was no statistical significance in the distribution between JE and NA groups, and the incidence of bone marrow oedema in this study was about 1.7%(26/1532).

## Conclusions

Our results demonstrated that joint sounds, joint pain, contracture and ADDWR were high risk factors for joint effusion, and the OR value of joint pain was as high as 8.463. Joint effusion reflects the possibility of non-bacterial inflammatory reaction or synovial circulation disorder in the TMJ. Because all the mentioned four risk factors can be alleviated with non-surgical treatment, joint effusion is not recommended as an indication for joint surgery.

## Data Availability

The datasets generated and/or analyzed during the current study are not publicly available due to ethical concerns but are available from the corresponding author on reasonable request.

## Abbreviations

TMD: Temporomandibular disorder
TMJ: Temporomandibular joint
MIO: Maximal interincisal opening
NA: Normal
PDD: Posterior disc displacement
PDDWR: Posterior disc displacement with reduction
PDDWoR: Posterior disc displacement without reduction
ADDWR: Anterior disc displacement with reduction
ADDWoR: Anterior disc displacement without reduction
MRI: Magnetic resonance imaging
ROC: Receiver operating curve
AUC: Area under the curve

## References

[1] Topaloglu Yasan G, Adiloglu S, Tuz HH, Sahar D (2023). Evaluation of clinical signs and magnetic resonance imaging findings in patients with temporomandibular disorders. J Craniomaxillofac Surg.

[2] Balel Y, Yildiz S, Gokce E, Tumer MK, Ege B (2023). Does Temporomandibular Joint magnetic resonance imaging diagnosis support clinical examination diagnosis following diagnostic criteria for Temporomandibular disorders?. J Oral Maxillofac Surg.

[3] Vogl TJ, Günther D, Weigl P, Scholtz JE (2021). Diagnostic value of dynamic magnetic resonance imaging of temporomandibular joint dysfunction. Eur J Radiol Open.

[4] Takahara N, Nakagawa S, Sumikura K, Yoda T (2023). Comparison of magnetic resonance imaging findings in patients with intermittent closed lock and acute closed lock of the temporomandibular joint: a cross-sectional retrospective study. Oral Radiol.

[5] Zhang J, Yu W, Wang J (2023). A comparative study of temporomandibular joints in adults with definite sleep bruxism on magnetic Resonance Imaging and Cone-Beam Computer Tomography images. J Clin Med.

[6] Li C, Zhang Q (2023). Comparison of imaging findings of 714 symptomatic and asymptomatic temporomandibular joints: a retrospective study. BMC Oral Health.

[7] Bouloux GF. The use of synovial fluid analysis for diagnosis of temporomandibular joint disorders [J].Oral Maxillofac Surg Clin North Am,2018,30(3):251–6. 10.1016/j.coms.2018.03.001 29861340

[8] Bayındır Ş, Yılmaz Asan C, Demirbaş AE, Keti DB, Kütük N (2022). Evaluation of aggrecan and adipokine levels in temporomandibular joint synovial fluid. J Craniomaxillofac Surg.

[9] Díaz Reverand S, Muñoz Guerra M, Rodríguez Campo J et al. Correlation between joint effusion and clinical symptoms, magnetic resonance imaging and arthroscopic findings in patients with temporomandibular joint disease. J Craniomaxillofac Surg. 2020;48(12):1146–1151. 10.1016/j.jcms.2020.10.003IF: 3.1 B2. 10.1016/j.jcms.2020.10.003 33199210

[10] Xu J, Wang D, Yang C, Wang F, Wang M (2023). Reconstructed magnetic resonance image-based effusion volume assessment for temporomandibular joint arthralgia. J Oral Rehabil.

[11] Higuchi K, Chiba M, Sai Y (2020). Relationship between temporomandibular joint pain and magnetic resonance imaging findings in patients with temporomandibular joint disorders. Int J Oral Maxillofac Surg.

[12] Hu YK, Yang C, Xie QY (2016). Changes in disc status in the reducing and nonreducing anterior disc displacement of temporomandibular joint: a longitudinal retrospective study. Sci Rep.

[13] Roh HS, Kim W, Kim YK, Lee JY (2012). Relationships between disk displacement, joint effusion, and degenerative changes of the TMJ in TMD patients based on MRI findings. J Craniomaxillofac Surg.

[14] Eriksen ES, Hellem S, Skartveit L (2020). Temporomandibular joint pain and associated magnetic resonance findings: a retrospective study with a control group. Acta Radiol Open.

[15] Hosgor H. The relationship between temporomandibular joint effusion and pain in patients with internal derangement [published correction appears in J Craniomaxillofac Surg. 2021;49(8):748]. J Craniomaxillofac Surg. 2019;47(6):940–944. 10.1016/j.jcms.2019.03.010. 10.1016/j.jcms.2019.03.010 30935852

[16] Manfredini D, Basso D, Arboretti R, Guarda-Nardini L (2009). Association between magnetic resonance signs of temporomandibular joint effusion and disk displacement. Oral Surg Oral Med Oral Pathol Oral Radiol Endodontol.

[17] Suenaga S, Nagayama K, Nagasawa T, Indo H, Majima HJ (2016). The usefulness of diagnostic imaging for the assessment of pain symptoms in temporomandibular disorders. Jpn Dent Sci Rev.

[18] Yap AU, Lei J, Zhang XH, Fu KY (2023). TMJ degenerative joint disease: relationships between CBCT findings, clinical symptoms, and signs. Acta Odontol Scand.

[19] Rezazadeh F, Esnaashari N, Azad A, Emad S (2022). The effects of botulinum toxin a injection on the lateral pterygoid muscle in patients with a painful temporomandibular joint click: a randomized clinical trial study. BMC Oral Health.

[20] Schiffman E, Ohrbach R, Truelove E (2014). Diagnostic criteria for Temporomandibular disorders (DC/TMD) for clinical and Research Applications: recommendations of the International RDC/TMD Consortium Network* and Orofacial Pain Special Interest Group†. J Oral Facial Pain Headache.

[21] Alshamaa A, Aldelaimi T (2020). Treatment of Myogenic Temporomandibular Joint disorders with Diode laser and pharmacotherapy (comparative study). Int Med J.

[22] de Almeida AM, Botelho J, Machado V, Mendes JJ, Manso C, González-López S (2023). Comparison of the efficacy of two protocol treatments in patients with symptomatic disc displacement without reduction: a Randomized Controlled Trial. J Clin Med.

[23] Dias GM, Grossmann E, Carvalho ACP, Devito KL, Dos Santos MF, Ferreira LA. MRI changes and clinical characteristics in temporomandibular joints with displacement of the articular disk without reduction - a cross-sectional observational study [published online ahead of print, 2023 Apr 25]. Cranio. 2023;1–10. 10.1080/08869634.2023.2203039. 10.1080/08869634.2023.2203039 37097122

[24] Liu SS, Xu LL, Liu LK, Lu SJ, Cai B (2023). Platelet-rich plasma therapy for temporomandibular joint osteoarthritis: a randomized controlled trial [published online ahead of print, 2023 Oct 7]. J Craniomaxillofac Surg.

[25] Koca CG, Gumrukcu Z, Bilgir E (2020). Does clinical findings correlate with magnetic resonance imaging (MRI) findings in patients with temporomandibular joint (TMJ) pain? A cross sectional study. Med Oral Patol Oral Cir Bucal.

[26] Luo D, Qiu C, Zhou R, Yu W, Li X, Yang J (2022). MRI-based observation of the size and morphology of temporomandibular joint articular disc and condyle in young asymptomatic adults. Dentomaxillofac Radiol.

[27] Amaral Rde O, Damasceno NN, de Souza LA, Devito KL (2013). Magnetic resonance images of patients with temporomandibular disorders: prevalence and correlation between disk morphology and displacement. Eur J Radiol.

[28] Li C, Zhang Q (2022). Comparison of magnetic resonance imaging findings in 880 temporomandibular disorder patients of different age groups: a retrospective study. BMC Oral Health.

[29] Afroz S, Naritani M, Hosoki H, Takechi K, Okayama Y, Matsuka Y (2018). Prevalence of posterior disc displacement of the Temporomandibular Joint in patients with Temporomandibular disorders: systematic review and Meta-analyses. J Oral Facial Pain Headache.

